# Objective evaluation of choroidal melanin loss in patients with Vogt–Koyanagi–Harada disease using polarization-sensitive optical coherence tomography

**DOI:** 10.1038/s41598-022-07591-9

**Published:** 2022-03-03

**Authors:** Masahiro Miura, Shuichi Makita, Yoshiaki Yasuno, Shinnosuke Azuma, Toshihiro Mino, Tatsuo Yamaguchi, Takuya Iwasaki, Rei Nemoto, Hiroyuki Shimizu, Hiroshi Goto

**Affiliations:** 1 Department of Ophthalmology, Tokyo Medical University, Ibaraki Medical Center, 3-20-1 Chuo, Ami, Inashiki, Ibaraki 300395 Japan; 2 Computational Optics Group, University of Tsukuba, Tsukuba, Japan; 3 Topcon Corporation, Tokyo, Japan; 4 Department of Ophthalmology, Tokyo Medical University, Tokyo, Japan

**Keywords:** Translational research, Uveal diseases, Imaging and sensing

## Abstract

In this study, sunset glow fundus was evaluated in patients with Vogt–Koyanagi–Harada (VKH) disease using polarization-sensitive optical coherence tomography (PS-OCT). We evaluated 40 VKH eyes (20 patients) and 59 healthy eyes (59 age-matched controls). VKH eyes were divided into three groups according to color fundus images: sunset (17 eyes), potential sunset (13 eyes), and non-sunset (10 eyes). Choroidal melanin thickness (ChMeT) and the choroidal melanin thickness ratio (ChMeTratio) were calculated based on the degree of polarization uniformity from PS-OCT. ChMeT was significantly lower in sunset eyes than in non-sunset or control eyes (P = 0.003). The ChMeTratios of sunset or potential sunset eyes were significantly lower than those of non-sunset or control eyes (P = 0.04). Regional evaluation of ChMeT and the ChMeTratio showed that choroidal depigmentation predominantly occurred in the macula’s outer ring area (P = 0.002). The areas under receiver operating characteristic curves discriminating combined sunset (sunset and potential sunset) from non-sunset eyes were 0.983 and 0.997 for ChMeT and the ChMeTratio, respectively. Time course evaluation of 12 eyes from disease onset showed that ChMeT and the ChMeTratio significantly decreased over time. PS-OCT may be useful for objectively evaluating choroidal depigmentation in patients with VKH disease.

## Introduction

Vogt–Koyanagi–Harada (VKH) disease is a systemic autoimmune condition that targets melanocyte-containing organs including the eyes, skin, meninges, and inner ear^1^. The clinical course of VKH disease follows four stages: prodromal, uveitis, convalescence, and recurrence/chronic disease^1^. In some patients in the convalescent or recurrence/chronic stage, choroidal depigmentation occurs following choroidal melanocyte damage and the fundus develops an orange-red discoloration called sunset glow fundus (SGF)^1^. The appearance of SGF is highly specific to VKH disease. Thus, SGF is an important part of the Revised Diagnostic Criteria for VKH disease^2^ and the classification criteria for VKH disease^3^. Associations between SGF appearance, disease severity, and chronic ocular inflammation have also been reported^4,5^. Therefore, clinical evaluation of the presence and development of SGF is crucial for clinical management of patients with VKH disease.

In clinical practice, diagnosis of SGF is usually performed by subjective evaluation of color fundus images^6^. Uncertainty introduced by this subjective approach may cause difficulties in diagnosis and grading of SGF^7^. Objective approaches based on the ratio of color balance (sunset glow index)^8^ and visibility of the choroidal vessels (choroidal vascular appearance index)^9^ have been developed based on color fundus images. However, these methods were unable to discriminate choroidal depigmentation from choroidal thinning^10,11^. In addition, our previous study showed substantial overlap between eyes with SGF and those without SGF using the sunset glow index^12^. These methods were not studied further and are rarely used in clinical practice.

Polarization-sensitive optical coherence tomography (PS-OCT) is a functional extension of OCT in which three-dimensional polarization images of the human eye are acquired in vivo^13^. Melanin in tissues, including the choroid, can scatter light, causing depolarization or polarization scrambling^14^. We previously reported that PS-OCT was useful for objective evaluation of choroidal depigmentation in eyes with SGF^12^. However, our previous study had several limitations. For instance, only three sets of B-scan images were used to evaluate each eye without volumetric measurements^12^. Heterogeneous choroidal depigmentation may be overlooked by measurement of a limited area of the choroid. Volumetric measurement is required to evaluate heterogeneity of choroidal depigmentation in different locations. Another limitation was that evaluation was performed at a single time point for each patient, with no time-course analysis^12^. Time-course analysis starting from disease onset is important to evaluate the development of SGF. To overcome these limitations, we performed volumetric analysis using PS-OCT in this study to evaluate the distribution of choroidal depigmentation in patients with SGF. The development of SGF was investigated using time-course analysis.

## Methods

### Patients

This prospective, observational, cross-sectional study was performed using a protocol that adhered to the tenets of the Declaration of Helsinki. Institutional Review Board approval was obtained from Tokyo Medical University (IRB 16-15, T2019-0072, and T2019-0217). The study was registered in the University Hospital Medical Information Network database (UMIN000026307, 000039650, and 000039648; http://www.umin.ac.jp/ctr/index-j.htm). The nature of the research and the implications of participating in the study was explained to all potential participants. Written informed consent was obtained from each participant before any study procedures or examinations were performed.

We examined 40 eyes from 20 Japanese patients (eight men and 12 women) with chronic VKH disease (Supplementary Table S1). Diagnosis of VKH disease was based on the Revised Diagnostic Criteria for VKH disease^2^. Among the study cohort, 12 eyes from six patients were in the chronic/recurrence phase, while the remaining 28 eyes from 14 patients were in the convalescent phase without recurrence. All patients were treated with intravenous high-dose corticosteroids followed by slowly tapered oral prednisolone. The mean duration from initial disease onset to PS-OCT measurement was 35.7 months (range, 8–95 months). The mean age at PS-OCT measurement was 48.9 years (range, 25–70 years). For six of the 20 patients with VKH disease, a follow-up study was performed from 3 to 18 months after disease onset (Supplementary Table S1). For these patients, PS-OCT data at 18 months after disease onset were combined with data from the single timepoint study for the full cohort of 20 patients with VKH disease.

We also evaluated 59 eyes from 59 age-matched healthy control Japanese participants (40 men and 19 women; age range, 23–70 years; mean age, 50.7 years). The right eye of each participant in the control group was evaluated. Exclusion criteria for the control group were history of intraocular surgery, retinal and/or choroidal pathology, or glaucoma. There were no significant differences between the ages of healthy controls and patients with VKH disease (P = 0.59; Mann–Whitney *U* test). Axial length was measured using an optical biometer (OA-2000, Tomey, Nagoya, Japan). The mean axial length of healthy control eyes was 24.5 mm (range, 22.2–27.9 mm) and that of the eyes of patients with VKH disease was 24.2 mm (range, 22.9–27.5 mm). There was no significant difference in axial length between the eyes of healthy controls and those of patients with VKH disease (P = 0.38; Mann–Whitney *U* test).

### Color fundus images

Color fundus images (1500 × 1500 pixels) with a 45° visual angle were captured using a built-in color fundus camera (DRI-OCT Triton; Topcon, Inc., Tokyo, Japan). Using these color fundus images, two ophthalmologists (R.N. and H.S.) subjectively rank-ordered the presence and appearance of SGF as (i) non-sunset, (ii) potential sunset, or (iii) sunset. Non-sunset was defined as color fundus images that did not show any findings suggestive of SGF. Potential sunset was defined as color fundus images showing the presence of diffuse faint retinal depigmentation or some degree of localized depigmentation. Sunset was defined as color fundus images showing diffuse apparent depigmentation. If discrepancies in the assignment of SGF occurred, a third ophthalmologist (I.T.) acted as referee until a consensus was reached.

**Figure 1:**
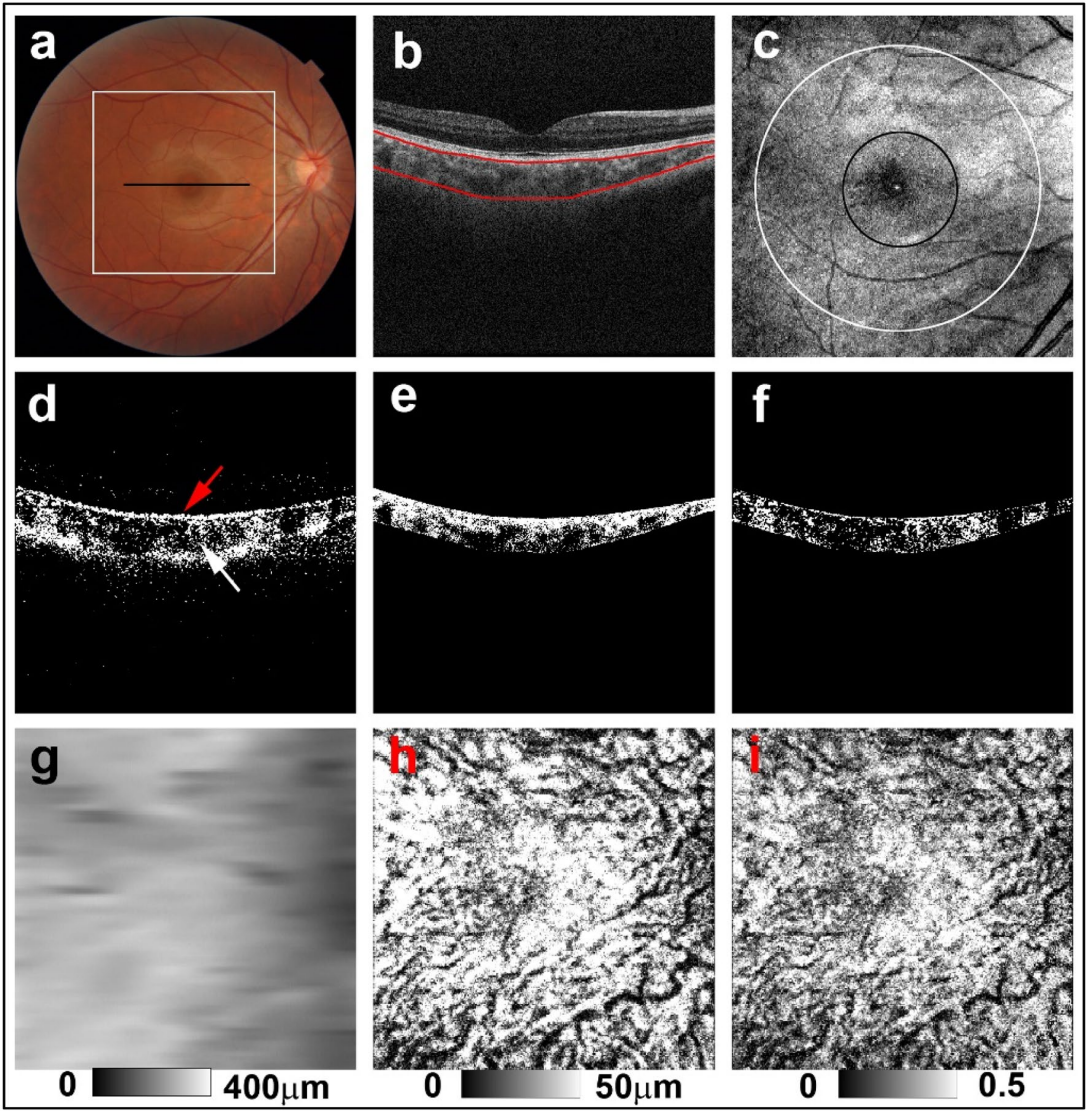
PS-OCT imaging of the right eye of a 34-year-old man from the healthy control group. (**a**) The black line in the color fundus image designates the scan line for PS-OCT B-scan images and the white line designates the area for the sunset glow index measurement. (**b**) Red lines in the standard OCT B-scan image were boundaries for automatic selection of the choroid area. (**c**) *En-face* projection image of standard OCT. The whole area was defined as the inner area of the outer circle (white line), the outer ring area was defined as the area between the outer circle (white line) and the inner circle (black line), and the center area was defined as the inner area of the inner circle (black line). (**d**) Binary DOPU B-scan image (DOPU < 0.8) showing distribution of RPE melanin (red arrow) and choroidal melanin (white arrow). (**e**) Binary B-scan image of choroidal interstitial stroma. (**f**) Binary B-scan image of a low DOPU area (< 0.8) within the choroidal interstitial stroma. (**g**) Choroidal thickness map, (**h**) ChMeT map, and (**i**) ChMeTratio map calculated using volumetric data from PS-OCT.

For objective evaluation of color balance in color fundus images without optic discs or peripapillary atrophy, square images with side lengths of 800 pixels were prepared (Fig. 1a). According to image analysis software (IMAGEnet, Topcon, Inc.), this was equivalent to an area of approximately 9 mm around the fovea. Three-color channel luminance was measured on a 256-step scale using Fiji image processing software^15^. The sunset glow index was calculated according to a previous study^8^ as follows: sunset glow index = L_red_/(L_red_ + L_green_ + L_blue_), where L_red_ represents the mean luminance of the red channel, L_green_ represents the mean luminance of the green channel, and L_blue_ represents the mean luminance of blue channel. The sunset glow index was calculated for eyes of patients with VKH disease and those of healthy controls.

### PS-OCT

A detailed description of the prototype PS-OCT system has been previously published^16^. The PS-OCT system is a multi-functional swept-source OCT device with a 1-μm wavelength band and polarization-diversity detection capability. This multi-functional OCT provides standard OCT, OCT angiography, and degree of polarization uniformity (DOPU) images from a single measurement. Standard OCT images were obtained by coherent composition of four repeated scans^16^. DOPU was calculated with Makita’s noise correction using a 3 × 3-pixel kernel^17^. The presence of low DOPU indicated depolarization by multiply melanin-scattered light^18^. DOPU B-scan images represent areas of low DOPU (< 0.8) in B-scan images (Fig. 1d). A raster scanning protocol with 512 A-lines × 256 B-scans covering a 6.0 × 6.0-mm region of the retina was used for volumetric scans. The depth range of each B-scan image was 2.8 mm and the depth resolution in the tissue was 6 μm. For quantitative measurements, transverse magnification of PS-OCT images was calibrated using a modification of Littman’s method^19^.

For volumetric evaluation of choroidal melanin, we calculated the thickness of melanin in the choroidal interstitial stroma. First, the choroid area in standard OCT B-scan images obtained from PS-OCT datasets was selected using an automatic segmentation method that was identical to the built-in program of a commercially available OCT device (DRI-OCT Triton) (Fig. 1b). Choroidal thickness was calculated according to the automatically selected choroid area. Standard OCT B-scan images were binarized using the local Otsu method^20^ followed by median filtering using Fiji image processing software^15^ to separate the choroidal area into vessel and interstitial areas (Fig. 1e). The area of low DOPU (< 0.8), defined as the total area of low DOPU within the choroidal interstitial stroma, was calculated from the B-scan DOPU images (Fig. 1f). This threshold value of low DOPU (< 0.8) was determined based on our previous PS-OCT study of choroidal melanin^12^. Choroidal melanin thickness (ChMeT) was calculated by counting the number of pixels of low DOPU (< 0.8) within the choroidal interstitial stroma in each A-line. The physical length of each pixel in the axial direction was 4.3 μm; physical length was used to evaluate ChMeT. The choroidal melanin thickness ratio (ChMeTratio) was calculated from the percentage area of low DOPU (< 0.8), defined as the occupancy of the low DOPU area within the choroidal interstitial stroma. Using volumetric data from PS-OCT, a ChMeT map, a ChMeTratio map, and a choroidal thickness map were generated (Fig. 1g–i). The volumetric scan was divided by the inner and outer circle centers on the fovea with diameters of 2 mm and 5 mm, respectively (Fig. 1c). The mean ChMeT, mean ChMeTratio, and mean choroidal thickness of the whole area was calculated for the inner area of the outer circle. For regional evaluation of ChMeT and ChMeTratio, we defined the “center area” representing the area within the center circle and the “outer ring” area between the inner and the outer circles. The ratios of the mean ChMeT and the mean ChMeTratio at the center area to those of the outer ring were calculated.

To assess reproducibility, we evaluated ChMeT, the ChMeTratio, and choroidal thickness of the whole area for nine eyes from nine healthy controls (seven men and two women; age range, 28–60 years; mean age, 42.8 years). The coefficient of variation was calculated using four repeated measurements for each participant.

## Results

**Figure 2:**
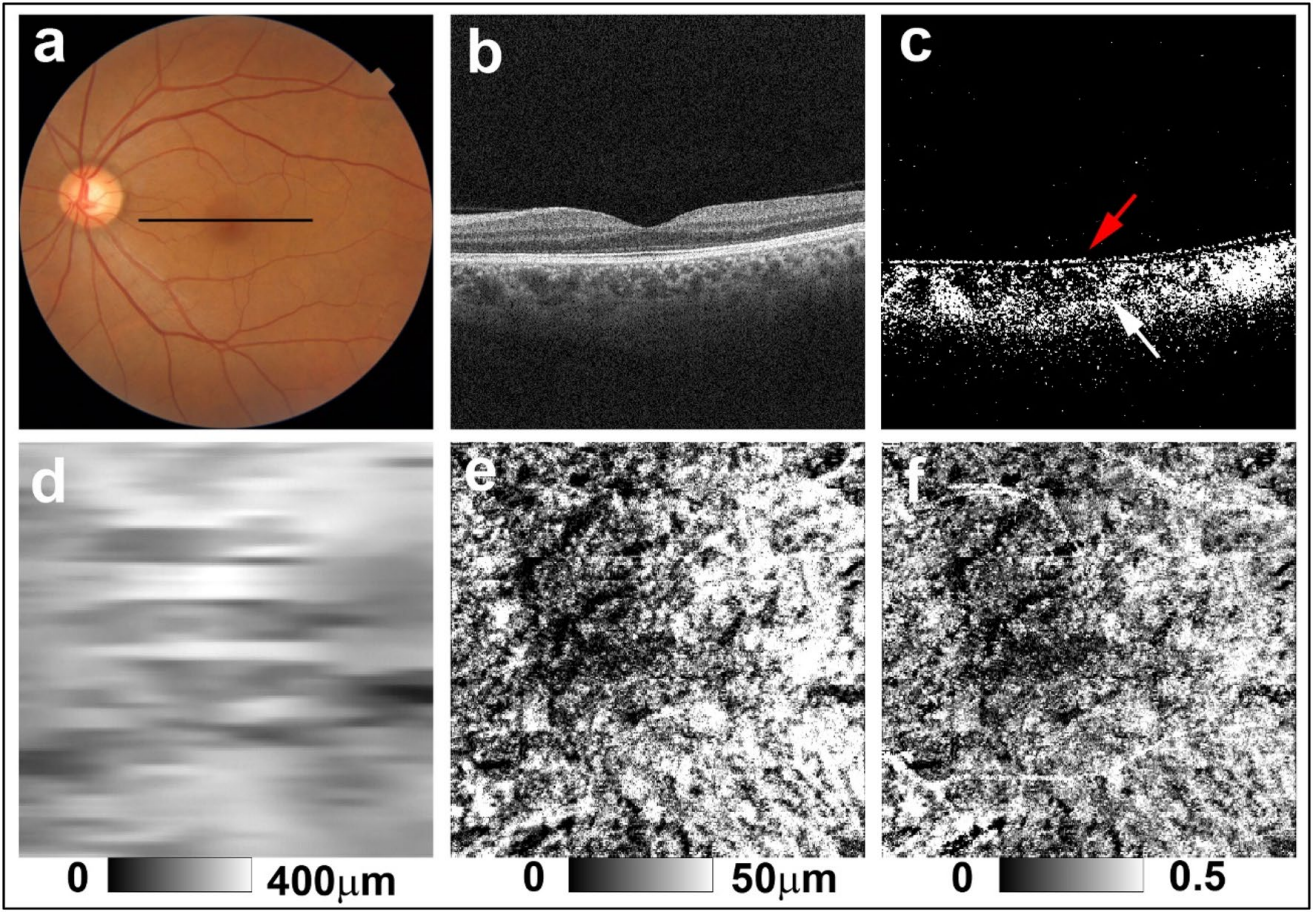
PS-OCT imaging of the left eye of a 56-year-old woman from the non-sunset group (Case 4 in Supplementary Table S1). (**a**) Color fundus images showed no evidence of sunset glow fundus. The black line in the color fundus image designates the scan line for PS-OCT B-scan images. (**b**) Standard OCT B-scan image. (**c**) Binary DOPU B-scan image (DOPU < 0.8) showing preservation of RPE melanin (red arrow) and choroidal melanin (white arrow). (**d**) Choroidal thickness map, (**e**) ChMeT map, and (**f**) ChMeTratio map.

**Figure 3:**
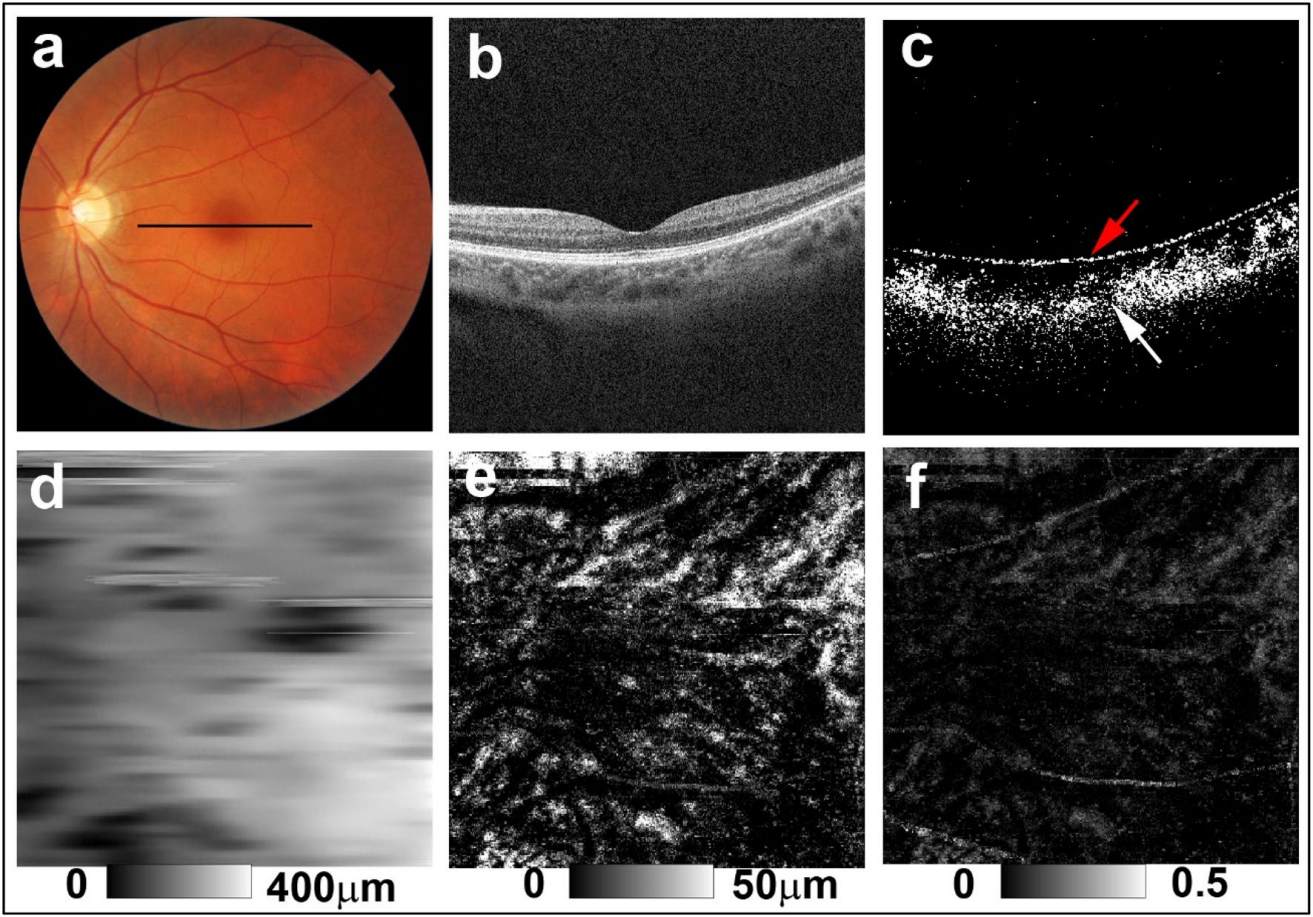
PS-OCT imaging of the left eye of a 43-year-old woman from the potential sunset group (Case 6 in Supplementary Table S1). (**a**) Color fundus images showed faint diffuse depigmentation. The black line in the color fundus image designates the scan line for PS-OCT B-scan images. (**b**) Standard OCT B-scan image. (**c**) Binary DOPU B-scan image (DOPU < 0.8) showing preservation of RPE melanin (red arrow) and mild reduction of choroidal melanin (white arrow). (**d**) Choroidal thickness map. (**e**) ChMeT map and (**f**) ChMeTratio map showing decreased choroidal melanin compared with healthy control eyes (Fig. 1) and non-sunset eyes (Fig. 2).

**Figure 4:**
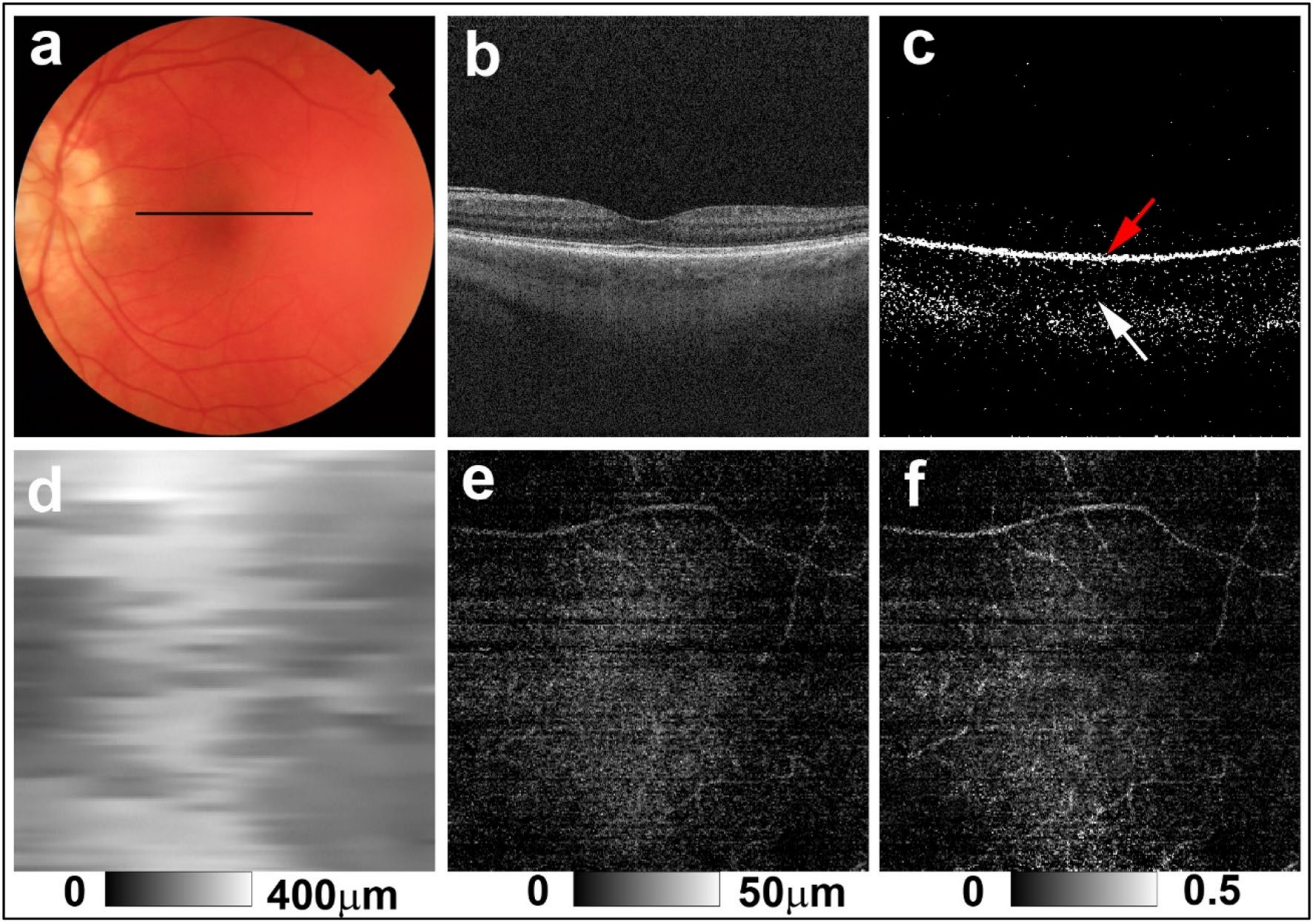
PS-OCT imaging of the left eye of a 49-year-old woman from the sunset group (Case 14 in Supplementary Table S1). (**a**) Color fundus image showing apparent diffuse depigmentation. The black line in the color fundus image designates the scan line for PS-OCT B-scan images. (**b**) Standard OCT B-scan image. (**c**) Binary DOPU B-scan image (DOPU < 0.8) showing preservation of RPE melanin (red arrow) and a clear reduction of choroidal melanin (white arrow). (**d**) Choroidal thickness map. (**e**) ChMeT map, and (**f**) ChMeTratio map showing decreased choroidal melanin.

Based on subjective evaluation of color fundus images of the eyes of patients with VKH disease, 10 eyes from six patients were classified as non-sunset, 13 eyes from nine patients were classified as potential sunset, and 17 eyes from 10 patients were classified as sunset. The weighted Kappa value for inter-observer agreement in assignment of non-sunset, potential sunset, and sunset based on color fundus images was 0.74. Figures 2, 3 and 4 show representative examples of non-sunset, potential sunset, and sunset eyes, respectively.

For the eyes of healthy controls, DOPU B-scan images showed depolarization consistent with retinal pigment epithelium (RPE) melanin and choroidal melanin (Fig. 1d). In non-sunset eyes, DOPU B-scan images showed preservation of choroidal melanin (Fig. 2c). Choroidal melanin content was decreased to some degree in potential sunset eyes (Fig. 3c) and was clearly decreased in sunset eyes (Fig. 4c). Regardless of SGF appearance, RPE melanin was preserved in the eyes of patients with VKH disease (Figs. 2c, 3c, and 4c).

**Figure 5:**
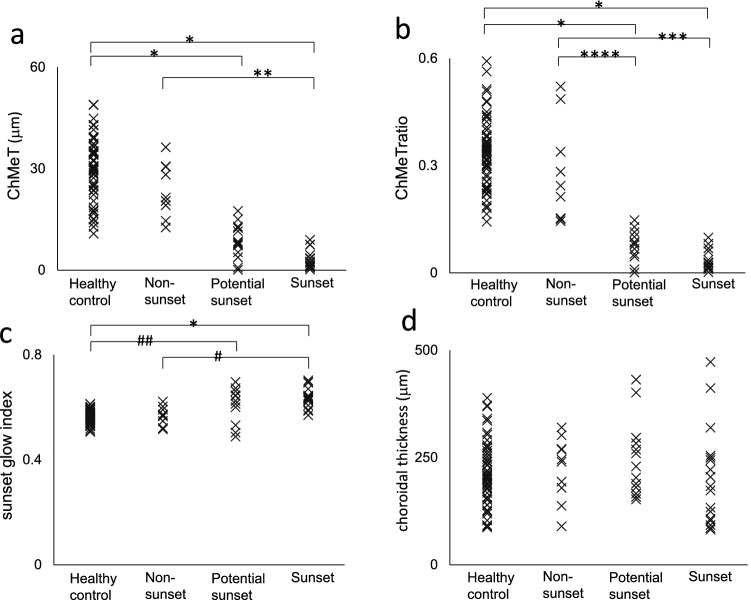
Distribution of ChMeT (**a**), the ChMeTratio (**b**), sunset glow index (**c**), and choroidal thickness (**d**) in healthy control, non-sunset, potential sunset, and sunset eyes. *P < 0.001, **P = 0.003, ***P = 0.002, ****P = 0.04, ^#^P = 0.001, ^##^P = 0.009.

For the objective evaluation, we compared ChMeT of the whole area, the ChMeTratio of the whole area, the sunset glow index, and choroidal thickness of the whole area among four groups of eyes (healthy control, non-sunset, potential sunset, and sunset) (Supplementary Table S2 and Fig. 5). The mean ChMeT of the whole area of sunset eyes was significantly smaller than those of non-sunset or control eyes (P = 0.003 and P < 0.001, respectively; Kruskal–Wallis test with Dunn’s post-hoc test). The mean ChMeT of the whole area of potential sunset eyes was significantly smaller than that of healthy control eyes (P < 0.001, Kruskal–Wallis test with Dunn’s post-hoc test). The mean ChMeTratio of the whole area of sunset eyes was significantly smaller than those of non-sunset or healthy control eyes (P = 0.002 and P < 0.001, respectively; Kruskal–Wallis test with Dunn’s post-hoc test). The mean ChMeTratio of the whole area of potential sunset eyes was significantly smaller than those of non-sunset or healthy control eyes (P = 0.04 and P < 0.001, respectively; Kruskal–Wallis test with Dunn’s post-hoc test). The mean sunset glow index of sunset eyes was significantly larger than those of non-sunset or healthy control eyes (P = 0.001 and P < 0.001, respectively; Kruskal–Wallis test with Dunn’s post-hoc test). The mean sunset glow index of potential sunset eyes was significantly larger than that of healthy control eyes (P = 0.009, Kruskal–Wallis test with Dunn’s post-hoc test). The mean choroidal thickness of the whole area showed no significant differences among the four groups of eyes (P = 0.41, Kruskal–Wallis test). The coefficients of variation of four repeated measurements (mean ± standard deviation) were 0.070 ± 0.045, 0.057 ± 0.039, and 0.017 ± 0.015 for ChMeT, the ChMeTratio, and choroidal thickness of the whole area, respectively.

**Figure 6:**
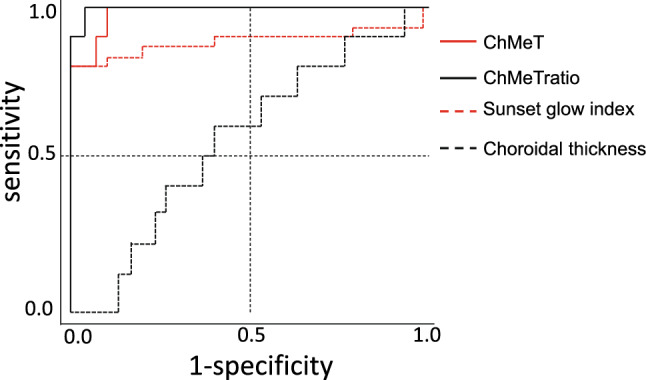
Receiver operating characteristic curves of ChMeT, the ChMeTratio, sunset glow index, and choroidal thickness for discrimination of eyes with choroidal depigmentation (30 eyes; sunset and potential sunset groups) and eyes without choroidal depigmentation (10 eyes; non-sunset group).

The abilities of ChMeT, the ChMeTratio, the sunset glow index, and choroidal thickness to discriminate between eyes with choroidal depigmentation and eyes without choroidal depigmentation were evaluated using receiver operating characteristic (ROC) curve analysis. Eyes of patients with VKH disease were divided into two groups: choroidal depigmentation (30 eyes; sunset and potential sunset groups) and non-sunset (10 eyes). The areas under the ROC curves (AUCs) were compared between these two groups. The AUCs were 0.983 (P < 0.001), 0.997 (P < 0.001), 0.843 (P < 0.001) and 0.557 (P = 0.58) for ChMeT, the ChMeTratio, the sunset glow index, and choroidal thickness, respectively (Fig. 6).

To evaluate regional differences, we compared ChMeT and the ChMeTratio at the center area with those of the outer ring area (Supplementary Table S3). In healthy control eyes, there were no significant differences between the mean ChMeT or mean ChMeTratio for these two areas (P = 0.85 and P = 0.06, respectively; Wilcoxon signed rank test). For the eyes of patients with VKH disease, mean ChMeT showed no significant difference between these two areas (P = 0.19, Wilcoxon signed rank test). The mean ChMeTratio at the center area was significantly larger than that at the outer ring area in the eyes of patients with VKH disease (P = 0.048, Wilcoxon signed rank test). For healthy control eyes, the ratio of the mean ChMeT at the center area to that of the outer ring area (center/outer ratio) showed no significant correlation with mean ChMeT of the whole area (R = − 0.142, P = 0.28). The center/outer ratio of the ChMeTratio also showed no significant correlation with the mean ChMeTratio of the whole area (R = 0.216, P = 0.10) (Supplementary Fig. S1). For the eyes of patients with VKH disease, the center/outer ratio of ChMeT showed a significant negative correlation with mean ChMeT of the whole area (R = − 0.619, P < 0.001). Moreover, the center/outer ratio of the ChMeTratio showed a significant negative correlation with the mean ChMeTratio of the whole area (R = − 0.417, P = 0.002) (Supplementary Fig. S1).

**Figure 7:**
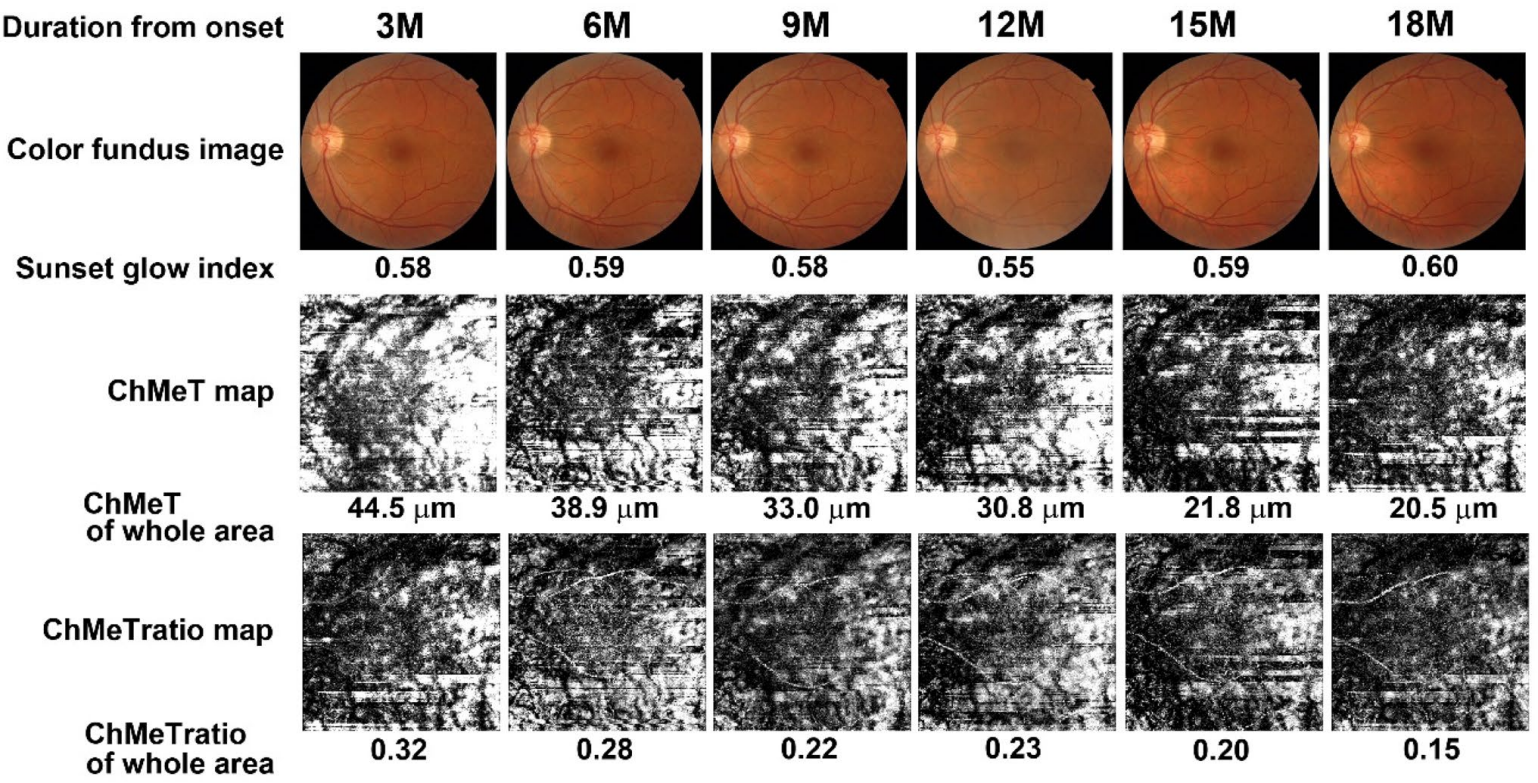
Time course analysis from 3 to 18 months after onset of Vogt–Koyanagi–Harada disease. Color fundus images, sunset glow index, ChMeT map, ChMeT of the whole area, ChMeTratio map, and ChMeTratio of the whole area are shown for the left eye of a 41-year-old man from the non-sunset group (Case 2 in Supplementary Table S1).

**Figure 8:**
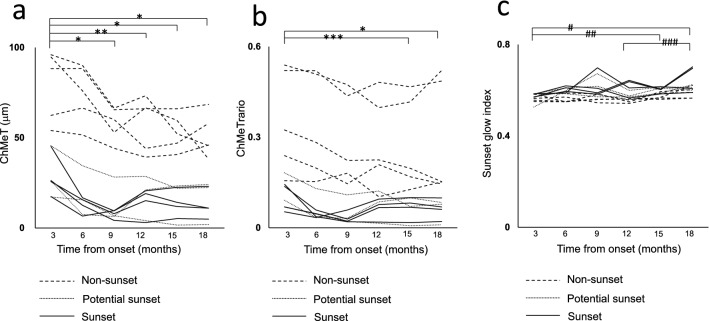
Time course analysis from 3 to 18 months after onset of Vogt–Koyanagi–Harada disease of ChMeT (**a**), the ChMeTratio (**b**), and sunset glow index (**c**). Time course analysis was conducted for 12 eyes (one line per eye). *P < 0.001, **P = 0.04, ***P = 0.004, ^#^P = 0.001, ^##^P = 0.034, ^###^P = 0.023.

To evaluate time-course changes in choroidal melanin, six patients with VKH disease (two men and four women; age range, 38–60 years; mean age, 47.5 years) were evaluated from 3 to 18 months after disease onset at 3-month intervals (Supplementary Table S4 and Fig. 7). Using subjective evaluation of color fundus images, five eyes from three patients were classified as non-sunset, three eyes from three patients were classified as potential sunset, and four eyes from three patients were classified as sunset. The mean ChMeT at 3 months after disease onset was significantly higher than that at 9, 12, 15, or 18 months after disease onset (P < 0.001, P = 0.04, P < 0.001, and P < 0.001, respectively; Friedman and Bonferroni post-hoc test) (Fig. 8a and Supplementary Table S4). The mean ChMeTratio at 3 months after disease onset was significantly higher than that at 15 or 18 months after disease onset (P = 0.004 and P < 0.001, respectively; Friedman and Bonferroni post-hoc test) (Fig. 8b and Supplementary Table S4). The mean sunset glow index at 3 months after disease onset was significantly lower than that at 15 or 18 months after disease onset (P = 0.034 and P = 0.001, respectively; Friedman and Bonferroni post-hoc test). The mean sunset glow index at 12 months after disease onset was significantly lower than that at 18 months after disease onset (P = 0.023, Friedman and Bonferroni post-hoc test) (Fig. 8c and Supplementary Table S4). For five non-sunset eyes, mean ChMeT and sunset glow index showed significant differences throughout the observation period (P = 0.02 and P = 0.012, respectively; Friedman test), but post-hoc tests did not show significant differences between individual assessments (P = 0.103 and P = 0.061, respectively; Bonferroni post-hoc test). The mean ChMeTratio showed significant differences throughout the observation period (P = 0.024, Friedman test), and post-hoc tests showed that the ChMeTratio at 3 months after disease onset was significantly larger than that at 15 months after disease onset (P = 0.024, Bonferroni post-hoc test) (Supplementary Table S5).

## Discussion

The appearance of SGF is a highly specific finding in VKH disease, and evaluation of SGF development is important for defining treatment strategies for VKH disease^2,3,5^. In current clinical practice, development of SGF is usually evaluated by subjective evaluation of color fundus images. This subjective approach may introduce uncertainty in patient evaluation and is a major obstacle to effective treatment. In this study, volumetric measurements of choroidal melanin were performed using PS-OCT. We confirmed the objective diagnostic capability of PS-OCT for appearance of SGF. PS-OCT was able to detect heterogeneity of choroidal depigmentation by location and was a useful follow-up evaluation tool for the development of SGF.

PS-OCT was able to evaluate the three-dimensional distribution of melanin in both the retina and choroid^12,13,21,22^. In normal chorioretinal tissue, melanin is found in choroidal melanocytes and RPE cells^23^. Histopathological studies of SGF showed that loss of melanin pigment occurred in choroidal melanocytes with preservation of RPE melanin^24–26^. To evaluate choroidal melanin loss in VKH disease, extraction of choroidal melanin in PS-OCT images is necessary to exclude the influence of RPE melanin. In our previous study, the area of the choroid was manually selected to measure choroidal melanin density^12^. This time-consuming manual procedure impedes volumetric evaluation; moreover, only three sets of B-scan images were used for each eye. In this study, we introduced automatic segmentation of choroidal melanin and conducted volumetric measurements using 256 B-scan images for each eye. This volumetric measurement enabled evaluation of regional differences of choroidal depigmentation.

In healthy control eyes, the center/outer ratio of the ChMeT showed no significant correlation with the mean ChMeT of the whole area. The center/outer ratio of the ChMeTratio also showed no significant correlation with the mean ChMeTratio of the whole area. For the eyes of patients with VKH disease, the center/outer ratio of the ChMeT showed a significant negative correlation with the mean ChMeT of the whole area. Moreover, the center/outer ratio of the ChMeTratio for the eyes of patients with VKH disease also showed a significant negative correlation with the mean ChMeTratio of the whole area. This finding indicated that choroidal depigmentation disproportionally occurred in the macula’s outer ring area. Regional differences in choroidal depigmentation might be an important indicator of localized choroidal inflammation and choroidal melanin damage. Further studies using wide field measurements will be required to evaluate the significance of regional differences in choroidal depigmentation.

Suzuki developed the sunset glow index for quantitative evaluation of color balance in color fundus photographs and reported its usefulness for evaluation of SGF development^8^. In this study, the sunset glow index showed limited ability to discriminate SGF appearance. In contrast, both ChMeT and the ChMeTratio showed high discriminatory ability for SGF appearance. There are several possible causes of the low discriminatory ability of the sunset glow index. One possibility is inconsistency in color balance in retinal color fundus photographs; in particular, red colors are frequently saturated in retinal images^27^. Another possibility is the influence of choroidal thinning: a negative correlation between sunset glow index and choroidal thickness has been previously reported^10^. Therefore, the influence of choroidal thickness should be considered in the evaluation of SGF using the sunset glow index. These limitations may influence the subjective evaluation of color fundus images, which is the current standard of clinical practice.

In this study, the ChMeTratio of eyes with potential sunset was significantly lower than that of non-sunset eyes and showed only a slight overlap with that of non-sunset eyes. This finding is consistent with most potential sunset eyes having choroidal depigmentation. In contrast, the sunset glow index of potential sunset eyes showed no significant difference compared with that of non-sunset eyes, with substantial overlap. The low discriminatory ability of the sunset glow index suggests that evaluation of color fundus photographs may overlook the presence of choroidal depigmentation. This problem is also expected to affect subjective evaluation of SGF using color fundus photographs, which is a widespread method in current clinical practice. Quantitative evaluation using PS-OCT might represent an important tool to address the uncertainty associated with SGF evaluation.

Continuous progression of choroidal depigmentation in VKH disease has been previously reported^8,9^. Progression is believed to be an important indicator of chronic choroidal inflammation. Our study also confirmed the progression of choroidal depigmentation via ChMeT, the ChMeTratio, and the sunset glow index. This progression was detected even in non-sunset eyes, which may represent progression of choroidal depigmentation even in eyes without SGF. Diagnosis of SGF could be made only in eyes with a certain degree of choroidal melanin reduction. The potential for choroidal melanocyte damage should be considered even in eyes without SGF.

In this PS-OCT study, both ChMeT and the ChMeTratio showed good discrimination of choroidal depigmentation, with very similar AUCs in ROC curve analysis (0.983 for ChMeT and 0.997 for the ChMeTratio). ChMeT represents the total volume of choroidal melanin and the ChMeTratio represent melanin density in the choroid. Both ChMeT and the ChMeTratio are good candidates for objective evaluation of SGF appearance. ChMeT showed no significant difference between potential sunset and non-sunset eyes, with substantial overlap. In contrast, the ChMeTratio showed significant differences between potential sunset and non-sunset eyes, with little overlap. This result suggested that the ChMeTratio may be a superior tool for evaluation of choroidal depigmentation. However, given the small numbers of patients in some groups in our study, we were only able to evaluate some aspects of VKH disease. Further studies of larger numbers of patients with VKH disease along with healthy controls and patients with other diseases will be required to fully evaluate the clinical utility of ChMeT and the ChMeTratio.

Our study had several limitations. First, given the small numbers of patients followed up over time, we could only evaluate some aspects of the time course of VKH disease. Second, we conducted follow-up analyses over 18 months from disease onset. Time-course analysis over a period of several years will be required to evaluate choroidal depigmentation, especially in non-sunset eyes. Third, this study only evaluated Japanese patients with VKH. Further studies of patients of other ethnicities will be required because of potential ethnic differences in choroidal melanin density^28^. Fourth, we used a threshold value for low DOPU (< 0.8) based on our previous PS-OCT study of choroidal melanin^12^. Although there is evidence supporting a monotonic relationship between DOPU and melanin pigmentation, the nature of this relationship is poorly understood. Further studies are required to accurately determine an appropriate threshold value for choroidal melanin measurement. Fifth, measurement of melanin thickness can be influenced by the kernel size of DOPU or melanin packing density in choroidal melanocytes. Therefore, neither ChMeT nor the ChMeTratio measure the actual thickness or density of choroidal melanin but are only proportional to the thickness or density of choroidal melanin, respectively. Sixth, melanin itself is a complex macromolecule consisting of eumelanin and pheomelanin^23,29^. Investigating the role of higher-order structures of choroidal melanin was beyond the capability of our PS-OCT measurements. Seventh, the physical mechanism of depolarization is incompletely understood. A potential additional mechanism of depolarization is scattering by non-spherical particles^30^. In this study, we assessed metrics derived from DOPU images to investigate choroidal melanin (ChMeT and the ChMeTratio). Many factors affect the DOPU, such as size of the spatial kernel to compute DOPU^31,32^, and the wavelength band used for OCT imaging^33^. These factors might have contributed to variation in the metrics used in this study. Special attention should be taken in comparing data for individual eyes obtained using different devices or conditions.

Another important limitation is the polarization state of incident light. DOPU measurements are known to highly dependent on the incident light polarization state^34–36^. Previous studies of DOPU, mainly performed at the Medical University of Vienna^14,18,31^, used a circularly polarized incident beam. In these cases, the incident polarization state-dependent DOPU variation would be minimal^35^, i.e., only variations in corneal and neural retinal birefringence among patients should be considered. In contrast, our PS-OCT used single-mode optical fibers in the sample arm^16^; hence, the polarization state of the incident beam to the eye was not fully stable and may vary among measurements. This suggests two limitations. First, our DOPU value may vary unpredictably among measurements and/or subjects. Second, the DOPU values obtained with our single-mode fiber-based PS-OCT^16^ cannot be directly compared with those from studies that used different PS-OCT systems^14,18,31^. Careful consideration is therefore needed when quantifying and generalizing our results. Nevertheless, our findings showed that metrics derived from binarized DOPU with our PS-OCT system have the potential to be used in the diagnosis of VKH disease and in monitoring choroidal melanin abnormalities. Further study is required to develop consistent regulation of polarization states for practical uses of PS-OCT systems.

In conclusion, this study demonstrated the clinical utility of PS-OCT imaging for quantitative evaluation of choroidal depigmentation in VKH disease. PS-OCT imaging facilitates the volumetric evaluation of choroidal melanin changes. Therefore, PS-OCT may represent an effective tool to characterize choroidal depigmentation in VKH disease.

## Supplementary Information

Supplementary Information.
