# Supplementary material for: Objective evaluation of choroidal melanin loss in patients with Vogt–Koyanagi–Harada disease using polarization-sensitive optical coherence tomography
Source: Sci Rep. 2022 Mar 3;12:3526. doi: 10.1038/s41598-022-07591-9 (PMC8894486; doi:10.1038/s41598-022-07591-9)
Supplement: Supplementary file 1 — Supplementary Information. [file 41598_2022_7591_MOESM1_ESM.pdf]

Objective evaluation of choroidal melanin loss in patients with Vogt–Koyanagi–Harada disease using polarization-sensitive optical coherence tomography

Masahiro Miura,[1*] Shuichi Makita,[2] Yoshiaki Yasuno,[2] Shinnosuke Azuma,[3] Toshihiro Mino[3], Tatsuo Yamaguchi[3], Takuya Iwasaki, [1] Rei Nemoto, [1] Hiroyuki Shimizu, [1] Hiroshi Goto[4]

[1] Department of Ophthalmology, Tokyo Medical University, Ibaraki Medical Center, Ami, Japan
[2] Computational Optics Group, University of Tsukuba, Tsukuba, Japan
[3] Topcon Corporation, Tokyo, Japan
[4] Department of Ophthalmology, Tokyo Medical University, Tokyo, Japan

* Corresponding author:
Masahiro Miura
Department of Ophthalmology, Tokyo Medical University, Ibaraki Medical Center
3-20-1 Chuo, Ami, Inashiki, Ibaraki 300395, Japan
Tel: +81-298-87-1161 Fax: +81-298-87-7656 E-mail: m-miura@tokyo-med.ac.jp

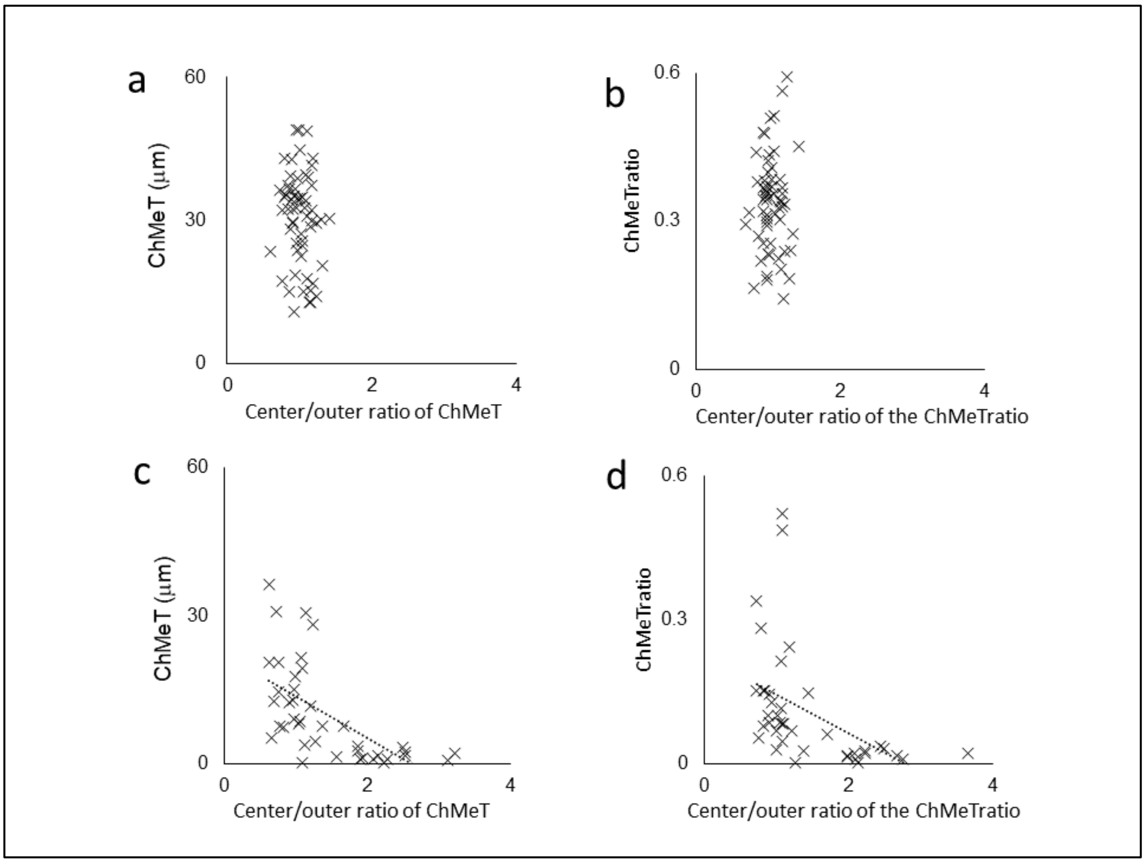

Supplementary Figure S1. Scatterplots illustrating the relationships between choroidal melanin thickness (ChMeT) and the center/outer ratio of ChMeT and between the choroidal melanin thickness ratio (ChMeTratio) and the center/outer ratio of the ChMeTratio. (a) Relationship between ChMeT and the center/outer ratio of ChMeT for the eyes of healthy controls. (b) Relationship between the ChMeTratio and the center/outer ratio of the ChMeTratio for the eyes of healthy controls. (c) Relationship between ChMeT and the center/outer ratio of ChMeT for the eyes of patients with Vogt–Koyanagi–Harada disease. The regression line is shown in black dashes for the correlation between ChMeT and the center/outer ratio of ChMeT. (d) Relationship between the ChMeTratio and the center/outer ratio of the ChMeTratio for the eyes of patients with Vogt–Koyanagi–Harada disease. The regression line is shown in black dashes for the correlation between the ChMeTratio and the center/outer ratio of the ChMeTratio.

Supplementary Table S1. Summary of the characteristics of patients with Vogt–Koyanagi–Harada disease enrolled int this study.

Case	Gender	Age (years)	Time since onset (months)	Classification		Phase of VKH disease	Participant in follow-up study?
				Right eye	Left eye		
1	Female	38	18	Non-sunset	Non-sunset	Convalescent phase	Yes
2	Male	41	18	Non-sunset	Non-sunset	Convalescent phase	Yes
3	Female	49	95	Non-sunset	Non-sunset	Convalescent phase	
4	Female	56	78	Non-sunset	Non-sunset	Convalescent phase	
5	Female	29	32	Potential sunset	Non-sunset	Convalescent phase	
6	Female	43	18	Non-sunset	Potential sunset	Convalescent phase	Yes
7	Female	25	48	Potential sunset	Potential sunset	Convalescent phase	
8	Female	37	43	Potential sunset	Potential sunset	Convalescent phase	
9	Male	70	21	Potential sunset	Potential sunset	Convalescent phase	
10	Female	41	8	Potential sunset	Potential sunset	Convalescent phase	
11	Female	30	8	Potential sunset	Sunset	Convalescent phase	
12	Female	54	18	Sunset	Potential sunset	Chronic/recurrent phase	Yes
13	Male	60	18	Sunset	Potential sunset	Convalescent phase	Yes
14	Female	49	18	Sunset	Sunset	Chronic/recurrent phase	Yes
15	Male	69	51	Sunset	Sunset	Chronic/recurrent phase	
16	Male	65	45	Sunset	Sunset	Convalescent phase	
17	Male	65	69	Sunset	Sunset	Chronic/recurrent phase	
18	Male	59	42	Sunset	Sunset	Chronic/recurrent phase	
19	Female	40	12	Sunset	Sunset	Chronic/recurrent phase	
20	Male	57	54	Sunset	Sunset	Convalescent phase	

VKH, Vogt–Koyanagi–Harada.

Supplementary Table S2. ChMeT of the whole area, ChMeTratio of the whole area, sunset glow index, and choroidal thickness of the whole area in healthy control, non-sunset, potential sunset, and sunset eyes.

Parameter	Group	N	Mean ± standard deviation (range)
ChMeT (μm)	Healthy control	59	30.5 ± 9.7 (10.8–49.0)
	Non-sunset	10	23.5 ± 7.7 (12.6–36.3)
	Potential sunset	13	8.5 ± 5.3 (0.1–17.5)
	Sunset	17	3.0 ± 2.7 (0.2–9.0)
ChMeTratio	Healthy control	59	0.337 ± 0.097 (0.143–0.592)
	Non-sunset	10	0.269 ± 0.140 (0.145–0.521)
	Potential sunset	13	0.077 ± 0.042 (0.001–0.148)
	Sunset	17	0.034 ± 0.027 (0.002–0.099)
Sunset glow index	Healthy control	59	0.562 ± 0.024 (0.506–0.613)
	Non-sunset	10	0.563 ± 0.028 (0.516–0.603)
	Potential sunset	13	0.611 ± 0.067 (0.488–0.696)
	Sunset	17	0.642 ± 0.035 (0.570–0.702)
Choroidal thickness (μm)	Healthy control	59	213.6 ± 70.8 (87.2–389.1)
	Non-sunset	10	224.6 ± 73.5 (89.7–320.1)
	Potential sunset	13	247.6 ± 89.3 (152.2–431.6)
	Sunset	17	203.7 ± 114.2 (81.9–472.6)

ChMet, choroidal melanin thickness; ChMeTratio, choroidal melanin thickness ratio.

Supplementary Table S3. ChMeT and ChMeTratio at the center area and the outer ring area of healthy control eyes and eyes of patients with Vogt–Koyanagi–Harada disease.

Parameter	Group	Region	Mean ± standard deviation (range)
ChMeT (μm)	Healthy control (N=59)	Center area	30.5 ± 10.7 (10.0–58.0)
		Outer ring	30.6 ± 10.2 (11.0–52.9)
	VKH disease (N=40)	Center area	9.9 ± 8.7 (0.1–34.1)
		Outer ring	9.9 ± 10.0 (0.1–38.5)
ChMeTratio	Healthy control (N=59)	Center area	0.346 ± 0.116 (0.135–0.751)
		Outer ring	0.331 ± 0.096 (0.133–0.600)
	VKH disease (N=40)	Center area	0.111 ± 0.119 (0.001–0.556)
		Outer ring	0.105 ± 0.121 (0.001–0.510)

ChMet, choroidal melanin thickness; ChMeTratio, choroidal melanin thickness ratio; VKH, Vogt–Koyanagi–Harada.

Supplementary Table S4. Time course of ChMeT, the ChMeTratio, and sunset glow index in 12 eyes of patients with Vogt–Koyanagi–Harada disease.

Time from onset	ChMeT (μm), mean ±SD (range)	ChMeTratio, mean±SD (range)	Sunset glow index, mean±SD (range)
3 months	22.1 ± 11.5 (6.6–44.5)	0.21 ± 0.17 (0.05–0.54)	0.57 ± 0.02 (0.53–0.59)
6 months	16.6 ± 13.3 (3.2–38.9)	0.17 ± 0.18 (0.03–0.52)	0.58 ± 0.03 (0.55–0.62)
9 months	13.5 ± 10.8 (1.5–33.0)	0.15 ± 0.16 (0.02–0.47)	0.60 ± 0.05 (0.55–0.70)
12 months	14.6 ± 10.2 (1.4–30.8)	0.16 ± 0.15 (0.02–0.48)	0.58 ± 0.03 (0.54–0.64)
15 months	12.9 ± 8.9 (0.5–30.5)	0.15 ± 0.15 (0.01–0.47)	0.59 ± 0.02 (0.56–0.59)
18 months	12.5 ± 9.0 (0.8–30.6)	0.16 ± 0.17 (0.01–0.52)	0.62 ± 0.04 (0.57–0.70)

ChMet, choroidal melanin thickness; ChMeTratio, choroidal melanin thickness ratio; SD, standard deviation.

Supplementary Table S5. Time course of ChMeT, ChMeTratio, and sunset glow index of five non-sunset eyes.

Time from onset	ChMeT (μm), mean ±SD (range)	ChMeTratio, mean±SD (range)	Sunset glow index, mean±SD (range)
3 months	32.5 ± 9.5 (6.6–44.5)	0.36 ± 0.17 (0.16–0.54)	0.57 ± 0.02 (0.55–0.58)
6 months	30.2 ± 7.9 (22.2–38.9)	0.33 ± 0.17 (0.15–0.52)	0.56 ± 0.02 (0.55–0.59)
9 months	24.7 ± 5.9 (18.3–33.0)	0.29 ± 0.15 (0.15–0.47)	0.57 ± 0.02 (0.55–0.58)
12 months	24.2 ± 7.4 (14.5–30.8)	0.28 ± 0.15 (0.10–0.48)	0.55 ± 0.01 (0.54–0.56)
15 months	21.9 ± 5.0 (19.0–30.5)	0.28 ± 0.15 (0.13–0.47)	0.58 ± 0.01 (0.56–0.59)
18 months	21.5 ± 5.7 (14.6–30.6)	0.29 ± 0.17 (0.15–0.52)	0.59 ± 0.02 (0.57–0.62)

ChMet, choroidal melanin thickness; ChMeTratio, choroidal melanin thickness ratio; SD, standard deviation.

Supplementary Table S6. Summary of age, ChMeT, ChMeTratio, and choroidal melanin thickness of healthy controls enrolled in this study.

Subject	Age (years)	ChMeT (μm)	ChMeTratio	Choroidal thickness (μm)	Sunset glow index
1	56	34.8	0.441	176.2	0.539
2	47	29.9	0.367	191.6	0.537
3	66	44.9	0.348	335.9	0.556
4	68	29.7	0.305	208.4	0.553
5	69	18.6	0.439	92.3	0.581
6	53	29.4	0.319	218.6	0.527
7	61	39.1	0.328	259.6	0.577
8	48	23.5	0.293	180.2	0.566
9	66	25.7	0.385	139.6	0.584
10	56	32.6	0.399	188.7	0.571
11	69	38.9	0.435	219.6	0.554
12	65	43.0	0.564	152.3	0.565
13	69	34.4	0.381	209.8	0.132
14	63	12.8	0.182	176.5	0.553
15	63	39.2	0.508	157.8	0.569
16	54	22.4	0.223	239.4	0.567
17	58	48.9	0.478	241.2	0.549
18	70	25.2	0.592	87.2	0.549
19	57	34.6	0.255	371.3	0.571
20	55	27.3	0.369	139.0	0.603
21	54	49.0	0.423	265.8	0.565
22	67	37.4	0.342	303.5	0.544
23	49	15.0	0.188	205.7	0.596
24	64	32.3	0.256	278.7	0.548
25	60	12.8	0.302	89.5	0.612
26	41	32.6	0.369	191.2	0.154
27	34	41.5	0.334	252.5	0.568
28	26	16.8	0.203	184.8	0.506
29	51	39.4	0.385	231.6	0.557
30	63	42.8	0.292	389.1	0.548
31	42	42.9	0.344	246.7	0.579

32	43	30.3	0.241	340.8	0.556
33	37	35.1	0.357	210.0	0.532
34	31	30.5	0.275	258.9	0.552
35	43	37.3	0.481	156.4	0.574
36	36	36.4	0.317	308.2	0.543
37	55	36.6	0.344	202.4	0.591
38	28	48.8	0.514	206.3	0.528
39	39	34.5	0.362	199.4	0.569
40	35	32.2	0.315	219.5	0.613
41	61	23.7	0.407	127.2	0.574
42	33	36.6	0.350	290.5	0.562
43	51	17.4	0.165	273.4	0.591
44	67	32.4	0.366	209.9	0.598
45	23	14.0	0.238	119.5	0.555
46	62	24.7	0.231	274.5	0.569
47	23	34.8	0.220	369.1	0.543
48	70	28.8	0.450	151.3	0.567
49	44	10.8	0.182	123.7	0.587
50	69	35.3	0.380	233.7	0.563
51	65	17.7	0.234	151.9	0.590
52	67	15.2	0.298	103.4	0.559
53	23	30.9	0.356	197.4	0.543
54	29	34.1	0.329	214.1	0.518
55	39	28.3	0.313	232.8	0.570
56	34	14.9	0.143	241.6	0.558
57	23	32.2	0.269	272.5	0.511
58	64	20.5	0.340	124.4	0.599
59	33	29.4	0.355	167.5	0.530

ChMet, choroidal melanin thickness; ChMeTratio, choroidal melanin thickness ratio.

Supplementary Table S7. Summary of ChMeT, ChMeTratio, and choroidal melanin thickness in eyes of patients with Vogt–Koyanagi–Harada disease.

Case	Eye	ChMeT (μm)	ChMeTratio	Choroidal thickness (μm)	Sunset glow index
1	R	21.5	0.521	89.7	0.565
1	L	30.6	0.486	137.4	0.566
2	R	14.6	0.145	240.1	0.591
2	L	20.5	0.153	301.9	0.603
3	R	28.3	0.244	270.1	0.568
3	L	19.2	0.213	193.8	0.572
4	R	36.3	0.339	245.2	0.516
4	L	30.7	0.283	268.3	0.521
5	R	7.3	0.101	152.2	0.532
5	L	12.6	0.151	179.1	0.548
6	R	20.5	0.152	320.1	0.622
6	L	7.6	0.078	229.3	0.625
7	R	3.8	0.046	180.3	0.613
7	L	5.3	0.053	203.0	0.661
8	R	11.5	0.148	165.9	0.488
8	L	15.1	0.128	283.1	0.503
9	R	8.5	0.067	268.6	0.696
9	L	12.9	0.086	296.0	0.673
10	R	17.5	0.114	431.6	0.643
10	L	12.5	0.086	401.3	0.645
11	R	0.1	0.001	189.0	0.696
11	L	0.2	0.002	183.1	0.694
12	R	2.1	0.021	221.7	0.608
12	L	0.8	0.010	158.3	0.613
13	R	9.0	0.099	249.5	0.590
13	L	8.0	0.084	259.5	0.600
14	R	7.5	0.061	319.5	0.702
14	L	7.6	0.069	244.0	0.696
15	R	1.5	0.037	103.1	0.627
15	L	0.8	0.021	81.9	0.670
16	R	0.8	0.013	133.3	0.631
16	L	1.6	0.030	107.0	0.639

17	R	1.1	0.027	92.6	0.570
17	L	1.3	0.029	85.9	0.633
18	R	2.3	0.021	204.9	0.644
18	L	0.7	0.010	173.2	0.636
19	R	3.6	0.016	411.7	0.586
19	L	3.4	0.016	472.6	0.624
20	R	4.5	0.082	124.0	0.634
20	L	2.6	0.027	254.6	0.640

ChMet, choroidal melanin thickness; ChMeTratio, choroidal melanin thickness ratio; L, left; R, right.
